# Recurrent Thrombotic Vasculopathy in a Former Cocaine User

**DOI:** 10.1155/2015/763613

**Published:** 2015-12-17

**Authors:** Preeti Jadhav, Hassan Tariq, Masooma Niazi, Giovanni Franchin

**Affiliations:** ^1^Bronx Lebanon Hospital Center, Department of Medicine, 1650 Selwyn Avenue, Suite No. 10C, Bronx, NY 10457, USA; ^2^Bronx Lebanon Hospital Center, Department of Pathology, 1650 Grand Concourse, Bronx, NY 10457, USA

## Abstract

We report a case of a 35-year-old female who presented to the emergency room (ER) complaining of a pruritic rash involving multiple areas of the body. She had a significant history of cocaine use in the past. She had first developed a similar rash in 2013 when she was diagnosed with cocaine-induced vasculitis. Her urine toxicology had been positive for cocaine in the past until July 2013. She was incarcerated and attended a drug rehabilitation program after which she quit cocaine use, which was consistent with negative urine toxicology on subsequent admissions. Further workup did not reveal any other, autoimmune or infectious, etiology of this clinical presentation. The patient underwent biopsy of the skin lesion that was consistent with thrombotic vasculopathy likely secondary to levamisole.

## 1. Introduction

Levamisole is an anthelmintic and immunomodulator drug, which has been used in cancer therapy, to treat various immunological renal diseases and to treat a number of skin diseases, including Behçet's disease. It works as a nicotinic acetylcholine receptor agonist that causes continued stimulation of the parasitic worm muscles, leading to paralysis [1, 2]. However, the drug was withdrawn from the human market in 1999 because of serious side effects including leukopenia, agranulocytosis, and skin vasculitis [1–3]. This drug lately has been increasingly used as an adulterant in cocaine sold in the United States and Canada. According to one survey done in 2009, approximately 70% of cocaine in the USA is contaminated with levamisole [3, 4]. Small vessel cutaneous vasculitis may occur following the use of cocaine adulterated with levamisole [1]. Patients often present with tender purpura on the ears and necrotic reticuloform purpura on the trunk or extremities. Lab abnormalities usually detected are anti-neutrophil antibodies (ANCA), antiphospholipid antibodies, leukopenia, or neutropenia. Skin biopsy findings are often suggestive of leukocytoclastic vasculitis and small vessel thrombosis [3–6]. Urine toxicology testing can confirm cocaine use provided the patient utilized cocaine in the preceding two to three days. Testing for levamisole in serum or urine is difficult due to the short half-life (5.6 hours) of levamisole [3].

## 2. Case Presentation

A 35-year-old female was transferred to the emergency department (ED) of our hospital from a drug rehabilitation center for necrotic skin lesions. Two days before this presentation, the patient had noticed an itching sensation in her left ear. Later a reddish black rash appeared on the left ear. The rash was painful and progressively got worse, involving the right ear, hands, and lower back.

She denied fever, arthralgia, insect bite, or recent travel. Her medical history was significant for seizure disorder, asthma, bipolar disorder, and polysubstance abuse. She denied any recent use of recreational drugs and attested that she last used cocaine 8 months prior to being incarcerated.

**Figure 1:**
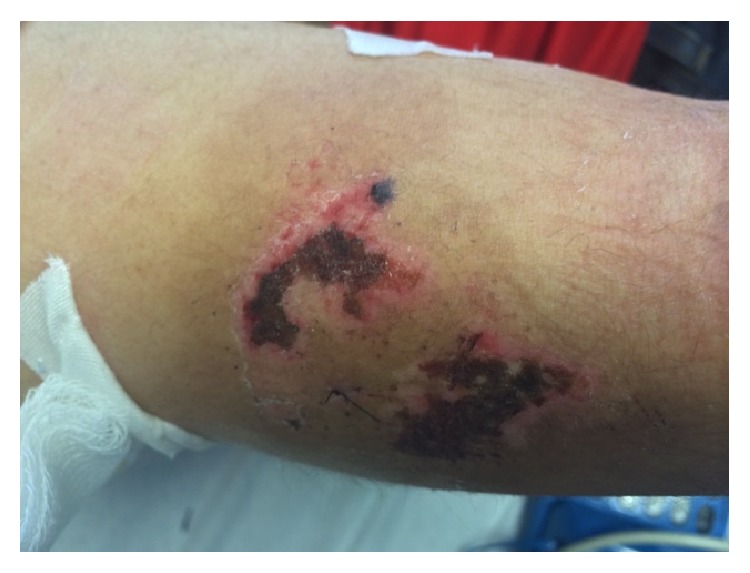
Multiple necrotic purple lesions on arms.

On examination patient was afebrile. Vital signs were noted as blood pressure 135/73 mm of hg, pulse rate of 95/min, and respiratory rate of 17/min, saturating 98% on room air. She was alert and oriented to time, place, and self. Precordial examination revealed normal heart sounds without any murmur or gallops. Auscultation of lungs revealed bilateral air entry without any adventitious sounds. There were multiple necrotic purple lesions on arms (Figure 1) bilateral pinnae, buttocks, and finger. All lesions had erythematous base, clear margins, no pus, or discharge.

The patient had a similar rash two years before this presentation and was diagnosed with levamisole-induced purpura as the workup to elucidate other etiologies that could have explained that the rash was unremarkable (Table 1). She had urine toxicology screening during each hospitalization that had been positive for cocaine and phenobarbital in the past (from 2011) but was subsequently negative since July 2014.

**Figure 2:**
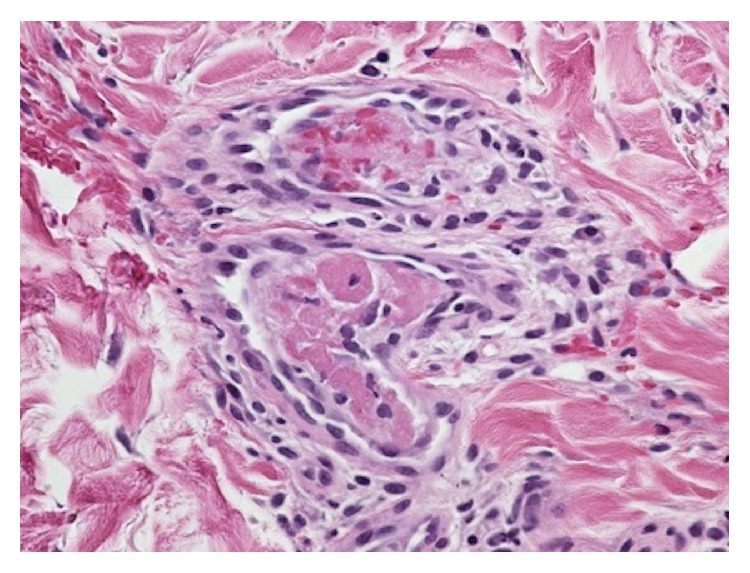
Thrombotic vasculopathy. High magnification showing occluded vessels with intraluminal fibrin and platelet thrombi (magnification 400x, H&E stain).

During the current admission the patient underwent biopsy of the skin lesion that showed several dermal vessels occluded by fibrin and platelet thrombi without signs of inflammation, consistent with thrombotic vasculopathy (Figure 2) likely secondary to levamisole.

She was treated with systemic steroids and discharged to rehabilitation center.

## 3. Discussion

Levamisole-induced vasculitis was first described in the 1970s [1]. This syndrome produces a characteristic clinical presentation of vasculitis in association with a variable pattern of immunologic disturbances. Cutaneous manifestations associated with levamisole use are varied and include nonspecific eruptions, lichenoid eruptions, fixed drug rash, and cutaneous vasculitis [4]. Lesions may appear suddenly and enlarge rapidly. Purpuric papules, plaques, hemorrhagic bullae, and even midline destruction have also been reported [7–9]. In the cocaine-levamisole cutaneous vasculopathy syndrome, lesions frequently have a distinctive morphology; they tend to be stellate with a bright erythematous border and necrotic appearing center [1, 2]. One of the most unique features of this syndrome is that the rash has a predilection for the ears, which may be due to the fact that the lower temperature of the ear may facilitate the deposition of immune complexes [10].

The syndrome has a very interesting spectrum of autoantibody findings. The immunological evidence is usually limited to presence of p-ANCA and sometimes c-ANCA antibodies [7, 8]. Anti-PR3 and anti-MPO are antibodies, respectively, associated with these ANCA patterns [1, 2].

The histology of cutaneous lesions typically shows thrombotic vasculitis or leukocytoclastic vasculitis with or without vascular occlusion [4–6]. The natural history of levamisole-induced vasculitis is spontaneous resolution without treatment when the levamisole is withdrawn. Immunologic abnormalities generally resolve within 2 to 14 months of withdrawal of the levamisole [2].

Detection of levamisole in both serum and urine must be performed using GC-MS or liquid chromatography-tandem mass spectrometry (LC-MS), as it is not detectable by routine toxicologic testing [11]. Clinical presentation and widespread adulteration of cocaine with levamisole are considered sufficient to make the diagnosis of this syndrome without any positive diagnostic test [10].

The important differential diagnosis to consider is Granulomatous Polyangiitis (GPA). In cocaine induced vasculitis, unlike GPA, granulomas and leukocytoclasia are not present histologically [9, 11–13]. In patients with cocaine-induced vasculitis, disease tends to be localized, whereas in patients with cutaneous GPA disease that was initially localized invariably progresses to be systemic, classically affecting organs such as the kidneys and lungs and the nasal cavity. Moreover, PR3 is more typical for vasculitis associated with cocaine use than GPA [9, 11–13].

Other most common causes of small- and medium-sized vasculitis like infectious, allergic, or drug related ones can be ruled out based on history and laboratory workup [11–14].

Review of articles from various sources showed that in cases reported to have cutaneous vasculitis associated with cocaine use, urine toxicology was positive for cocaine indicating active cocaine use. These lesions usually resolve spontaneously within a few weeks of drug discontinuation and recur with subsequent contaminated cocaine abuse.

We report a unique case where the patient presented with typical cutaneous vasculitis presumably from prior exposure to levamisole/cocaine however in abstinence for at least 8 months prior to current vasculitis flare. We propose that cocaine/levamisole use may trigger an abnormal immune response that may develop with clinical cutaneous vasculitis in the future even in the absence of new exposure to the drug.

## Figures and Tables

**Table 1:** Laboratory workup.

Labs (reference normal range)	Year 2011	Year 2015 (Jan.)
WBC (4.8–10.8 k/*μ*L)	1.9	5.9

ANC (1.5–8 k/*μ*L)	0.6	4.6

B2 microglobulin (0.8–2.2 mg/L)		1.3

Myeloperoxidase MPO (P-ANCA) (<1.0)	<1	<0.1

Proteinase PR3 (C-ANCA) (<1.0)	18	1.2

C3 level (90–150 mg/dL)		91

C4 level (16–47 mg/dL)		<7

ESR (0–30 mm/hr)	34	60

CRP (≤5.5 mg/L)		166

ANA	Negative	Negative

Neutrophil Ab	Detected	
